# Oat Fiber Alleviates Loperamide-Induced Constipation in Mice by Modulating Intestinal Barrier Function

**DOI:** 10.3390/nu17152481

**Published:** 2025-07-29

**Authors:** Yufei Shi, Yuchao Han, Jie Jiang, Di Wang, Zhongxia Li, Guiju Sun, Shaokang Wang, Wang Liao, Hui Xia, Da Pan, Ligang Yang

**Affiliations:** 1 Key Laboratory of Environmental Medicine and Engineering of Ministry of Education, Department of Nutrition and Food Hygiene, School of Public Health, Southeast University, No. 87, Dingjiaqiao, Gulou District, Nanjing 210009, China; h0107syf@163.com (Y.S.);; 2 BYHEALTH Institute of Nutrition & Health, Guangzhou 510700, China

**Keywords:** oat fiber, constipation, gut microbiota, gastrointestinal regulatory peptides, immune inflammatory response

## Abstract

**Objective:** To investigate the effects of oat fiber on animal constipation and elucidate its underlying mechanisms. **Methods:** Male BALB/c mice were randomly allocated into five groups: control group (CON), model control group (MODEL), low dose group (LOW), middle dose group (MIDDLE), high dose group (HIGH). Constipation was induced in the mice by intragastric administration of loperamide. Subsequently, the mice (except those in the CON and MODEL groups) were administered oat fiber intragastrically for 21 consecutive days. **Results:** Compared with the MODEL group, oat fiber significantly increased the number of fecal pellets, fecal wet weight, and fecal water content (*p* < 0.05), shortened the time to first black stool excretion (*p* < 0.05), and enhanced the small intestinal propulsion rate in constipated mice. Additionally, oat fiber significantly upregulated motilin (MTL) and gastrin (GAS) levels (*p* < 0.05), while downregulating vasoactive intestinal peptide (VIP) and somatostatin (SS) levels (*p* < 0.05). It also significantly reduced the transcription level of Aquaporin 8 (AQP8) (*p* < 0.05), effectively alleviating intestinal mucosal injury and immune inflammation. The relative expression levels of TNF-α and IL-1β were significantly decreased in the oat fiber group (*p* < 0.05). Gut microbiota analysis revealed that oat fiber increased both the abundance and diversity of gut microbiota in constipated mice. Specifically, oat fiber was found to enhance the relative abundance of Firmicutes while reducing that of *Bacteroidetes*. At the genus level, it promoted the proliferation of Lachnospiraceae_NK4A136_group and Roseburia. **Conclusions:** Oat fiber alleviates constipation in mice by modulating gastrointestinal regulatory peptides, gut microbiota, aquaporin and mitigating intestinal barrier damage and immune-inflammatory responses.

## 1. Introduction

As a common gastrointestinal disorder, constipation is characterized by specific intestinal symptoms, including difficult defecation, reduced defecation frequency, and dry stools [1]. According to worldwide surveys, the global prevalence of constipation is estimated at approximately 14% [2]. In recent years, the incidence of chronic constipation has been on the rise, driven by shifts in dietary habits, a faster-paced lifestyle, and the combined effects of social, environmental, and psychological factors. Statistics indicate that in China, the prevalence of constipation among elderly adults aged 60 to 110 years was significantly higher than in the general population, reaching 33.5% [3]. Additionally, women were more prone to chronic constipation than men, with a prevalence nearly twice as high, which imposed significant negative effects on patients’ daily lives and quality of life [4].

Dietary fiber (DF) was characterized by its porous structure and low density, which enabled it to effectively shorten intestinal transit time, increase fecal volume, and prevent or alleviate constipation through particle formation and water retention [5]. The solubility, viscosity, and fermentability of DF influenced the distribution of molecules in the gut and the metabolism of microorganisms [6]. DF had been recommended as a foundational treatment for chronic constipation. It could promote the secretion of intestinal mucus, increase stool volume, accelerate colonic transit, stimulate the growth of intestinal microbiota, and enhance the accumulation of bacterial fermentation products [7].

In recent years, oats have gained popularity as a health food due to their richness in biologically active compounds that were beneficial to human health. However, the mechanisms by which oats alleviated constipation, particularly through dietary fiber, remain underexplored. The effects of oat fiber on gastrointestinal regulatory peptides, intestinal aquaporins, inflammatory responses, and gut microbiota were studied in mice. Based on these findings, we examined the mechanism by which oat fiber alleviated constipation and provided a theoretical foundation for the advancement and practical utilization of commercial dietary fibers.

## 2. Materials and Methods

### 2.1. Materials and Instruments

Dietary fiber products were provided by BY-HEALTH Co., Ltd. (Guangzhou, China); ELISA kit, Weyou Te Bio-Technology Center (Nanjing, China); DNA extraction kit, Shanghai Ouyi Biomedical Technology Co., LTD. (Shanghai, China); HE staining, Wuhan Bolf Biotechnology Co., LTD. (Wuhan, China); Distilled water for gavage, Shanghai Suitian Environmental Protection Technology Co., LTD. (Shanghai, China); Loperamide hydrochloride, Xi’an Janssen Pharmaceutical Co., LTD. (Xi’an, China); Acacia gum powder, Qiyan Chemical Products (Cangzhou, China); Activated carbon, Weihui Shengyang Chemical Co., LTD. (Xinxiang, China); Thirty SPF male BALB/c mice were purchased from Shanghai Slake Laboratory Animal Co., LTD. (Shanghai, China) (License number: SCXK (Shanghai) 2022-0004).

### 2.2. Experimental Methods

#### 2.2.1. Solution Preparation

Ink formulation: Precisely measure 100 g of gum arabic and dissolve it in 800 mL of distilled water through boiling, continuing until the mixture achieves a clear and transparent appearance. Subsequently, add 50 g of activated carbon and boil the mixture three times to prepare the activated carbon solution. After cooling the solution to room temperature, adjust the volume to 1000 mL with purified water. Store the solution at 4 °C, and shake well before each use.

#### 2.2.2. Grouping of Animals

**Figure 1:**
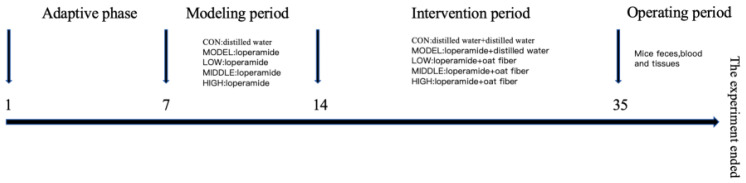
The whole process of animal experiment.

Male BALB/c mice weighing 20 ± 2 g were maintained in a controlled environment at 25 ± 2 °C with 50% ± 5% humidity and a 12-h light-dark cycle. The experimental period spanned 31 days, preceded by a 7-day acclimatization phase before the formal initiation of the study. In the design of animal experiments, the sample size for multiple groups was calculated using the sample size formula for one-way ANOVA, which is presented as follows. The definitions of the parameters are described below: $Z_{1-\alpha /2}$ represents the critical value of significance level (when α = 0.05, it is approximately 1.96); $Z_{1-\beta }$ represents the critical value of power (when power = 0.8, it is approximately 0.84); f represents the effect size (calculated based on the main indicators of the pre-experiment); k represents the number of groups. Based on the fundamental formula outlined below the primary experimental indicators are anticipated to yield statistically significant outcomes, with each group comprising approximately 6 mice. A total of 30 mice were randomly assigned to five experimental groups (*n* = 6 per group): control group (CON), model control group (MODEL), low-dose treatment group (LOW), medium-dose group (MIDDLE), and high-dose group (HIGH). During the modeling phase from day 1 to day 7, the CON group received 0.25 mL distilled water by gavage at 13:00 daily, while the MODEL and dose groups received 0.25 mL loperamide hydrochloride solution (4 mg/kg BW) at the same time. On the 8th day, oat fiber intervention was initiated. At 1:00 p.m. each day, both the MODEL group and the DOSE groups received intragastric administration of 0.25 mL of a 4 mg/kg·BW loperamide hydrochloride solution. One hour later, the CON group and the MODEL group were administered 0.25 mL of distilled water intragastrically. The three DOSE groups received 0.25 mL of oat fiber suspension intragastrically. Specifically, the doses of the LOW group, MIDDLE group, and HIGH group were 10 times, 15 times, and 20 times the recommended dose for the human body, respectively (calculated based on a standard body weight of 60 kg). The recommended human dose was 800 mg/day, then the daily dietary fiber intake for mice in each group was as follows: 132 mg/kg·BW for the LOW group, 198 mg/kg·BW for the MIDDLE group, and 264 mg/kg·BW for the HIGH group. Intragastric administration was performed continuously for 21 days. After the intervention, the mice were anesthetized with isoflurane to minimize pain. Subsequently, blood specimens were obtained from the orbital venous sinus of the experimental mice, followed by additional experimental procedures including tissue collection. All samples were processed and stored appropriately in accordance with standard laboratory (Figure 1).

$$n\;=\;\frac{2\;*\;(Z_{1-\alpha /2}+Z_{1-\beta })2}{f^{2}\;*\;k}\;+\;1$$

#### 2.2.3. Determination of Fecal Water Content

Feces were collected from 13:00 to 16:00 on the first day post-intervention, and the number of fecal pellets and wet weight for each mouse at different time points were recorded. Subsequently, the feces were dried at 65 °C until constant weight, and the dry weight was recorded after water loss.

$$\mathrm{Fecal}\;\mathrm{water}\;\mathrm{content}\;(\%)=\frac{Wet\;weight\;of\;feces-Dry\;weight\;of\;feces}{Wet\;weight\;of\;feces}\;\times \;100$$

#### 2.2.4. Determination of the Time of First Melena

At 13:00 on day 30, the mice in the control group received 0.25 mL of distilled water through oral gavage, whereas the remaining four groups were given 0.25 mL of loperamide hydrochloride solution using the same administration technique. After one hour, all animals were administered 0.25 mL of an activated carbon suspension via gavage, with the exact time of dosing documented. The fecal color was then closely observed, and the moment of the first appearance of melena (black stool) was noted. The interval before the onset of the first black stool was determined by subtracting the time of activated carbon administration from the time when melena was first observed.

#### 2.2.5. Determination of the Intestinal Propulsive Rate

After 12 h of food and water deprivation, mice were subjected to gavage at 13:00 on day 31. Mice in the CON group received 0.25 mL of distilled water, while the other four groups were administered 0.25 mL of loperamide hydrochloride solution. Thirty minutes later, all mice were gavaged with 0.25 mL of activated carbon suspension. Blood was collected via orbital puncture, and the mice were euthanized immediately 30 min thereafter. The complete small intestine was removed, with its proximal end aligned to the starting point of a ruler. The movement distance of the leading edge of the activated carbon mixture was noted as the “activated carbon propulsion length.”

$$\mathrm{Small}\;\mathrm{intestine}\;\mathrm{propulsion}\;\mathrm{rate}\;(\%)=\frac{Ink\;advance\;length\;(cm)}{Total\;length\;of\;the\;small\;intestine\;(cm)}\;\times \;100$$

#### 2.2.6. Determination of Serum Gastrointestinal Hormone Levels

Take out 50 μL of serum respectively from the −80 °C refrigerator. The levels of MTL, GAS, SP, SS, VIP, and 5-HT were measured in accordance with the protocols provided by the respective ELISA kits. Finally, the standard curve was obtained by the external standard method, and the concentration of the analyte in the sample was calculated based on the absorbance.

#### 2.2.7. HE Staining Method of Small Intestine

Deparaffinization and water washing of paraffin sections. Subsequently, hematoxylin and eosin (H&E) staining was carried out, followed by slide dehydration and mounting. The evaluation of colonic tissue lesions was primarily based on the morphological features and structural integrity of epithelial cells, goblet cells, and the mucosal layer thickness.

#### 2.2.8. Determination of Intestinal Aquaporin-Related Gene Transcription Levels

The mRNA expression levels of target genes in colonic tissues were analyzed using real-time fluorescence-based quantitative polymerase chain reaction (RT-qPCR). Total RNA was isolated from mouse colon tissues via the TRIzol reagent method, and complementary DNA (cDNA) was synthesized through reverse transcription (Table 1). Subsequently, fluorescent quantification was conducted using the CFX384 Real-Time PCR Detection System.

In this paper, the expression of AQP4 and AQP8 genes in colon tissue was determined. The primer sequences were shown in Table 2.

#### 2.2.9. Determination of Gut Microbiota in Mice

The V3-V4 region of the 16S rRNA gene (primers: 343F: 5′-TACGGRAGGCAGCAG-3′, 798R: 5′-AGGGTATCTAATCCT-3′) was amplified by PCR using bacterial DNA as the template. The PCR amplification was carried out in a total reaction volume of 50 μL, under the following thermal cycling parameters: an initial denaturation step at 94 °C for 5 min; followed by 30 cycles consisting of denaturation at 94 °C for 30 s, annealing at 50 °C for 30 s, and extension at 72 °C for 30 s; a final extension at 72 °C for 10 min; and a holding temperature of 12 °C for 10 min. Upon completion of the reaction, the amplified products were separated via electrophoresis on a 1.5% agarose gel. The desired DNA fragment of approximately 250 base pairs was excised from the gel and purified using a commercial gel extraction kit. Library preparation was performed in accordance with the supplier’s guidelines, and sequencing was conducted using the Illumina NovaSeq6000 (Illumina Inc., San Diego, CA, USA) to obtain high-throughput sequence reads. Fecal microbiota data derived from mouse samples were processed using the QIIME 2 pipeline, and in-depth analysis of gut microbial community composition was carried out through a cloud-based analytical platform.

### 2.3. Data Processing

Statistical analyses were carried out with SPSS version 27. The data displayed in the tables are expressed as mean ± standard deviation (SD). Inter-group differences were evaluated through one-way ANOVA, and Duncan’s multiple range test was applied for post hoc pairwise comparisons. GraphPad Prism 10.2.1 was used for data visualization, and *t*-tests were employed for analyzing differences between two groups.

## 3. Results

### 3.1. Effects of Oat Fiber on Body Weight in Mice

After the initiation of modeling, the trend of weight gain in the mice decreased slightly, and the weight loss was more pronounced compared to the control group (CON) (Table 3), which was consistent with previous studies [8]. Body weight change was one of the key indicators for evaluating constipation animal models, and the reduction in weight gain might be attributed to the administration of loperamide via gavage [9]. Gavage of oat fiber could mitigate this downward trend. Following intervention, mice in the oat fiber treatment groups exhibited an upward trend, showing a marked difference in comparison to the model control group (MODEL) (*p* < 0.05).

### 3.2. Effects of Oat Fiber on Defecation in Mice

Compared with the CON group, the MODEL group exhibited a significant reduction in the number of fecal pellets at 3 h (82.65%, *p* < 0.001) (Table 4). In comparison to the MODEL group, all dose-treated groups exhibited a statistically significant elevation in the number of fecal pellets excreted within 3 h (*p* < 0.001). The fecal water content in the MODEL group was significantly lower than that in the CON group (*p* < 0.001). Compared with the MODEL group, the fecal water content in the three dose groups increased significantly (*p* < 0.001) (Table 4). The time to the first black stool excretion was significantly prolonged in the MODEL group compared with the CON group (*p* < 0.001). Compared with the MODEL group, the three dose groups exhibited a significantly shorter time to the first black stool excretion (*p* < 0.001) (Table 4).

As shown in Table 4, compared with the CON group, the MODEL group demonstrated a significant decrease in the number of fecal pellets at 3 h, fecal wet weight, and fecal water content (*p* < 0.001), as well as a significant increase in the time to the first black stool excretion (*p* < 0.001). These results indicated that loperamide hydrochloride successfully induced reduced defecation and constipation-like symptoms in mice, confirming the successful establishment of the mouse constipation model. Oat fiber alleviated constipation, which was consistent with previous findings [10]. Additionally, the greater the fiber content in oats, the more pronounced the alleviation of constipation symptoms.

### 3.3. Effect of Oat Fiber on Small Intestinal Propulsion Rate in Mice

**Figure 2:**
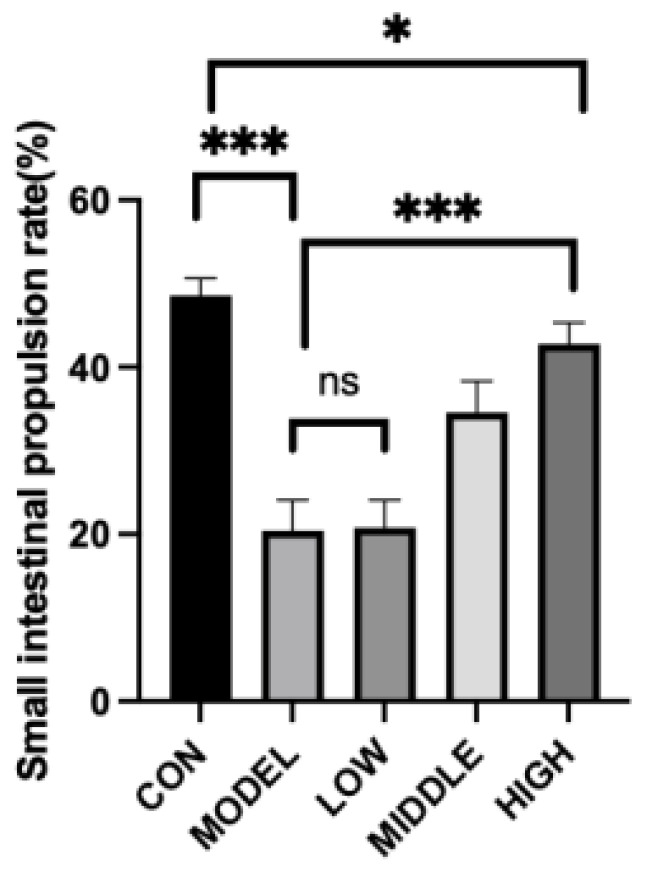
Effect of oat fiber on small intestinal propulsion rate in mice. Note: *, ***, denoted *p* < 0.05, *p* < 0.001, respectively. “ns” indicates that there was no significant difference.

Mice in the control group (CON) displayed normal intestinal motility, whereas the small intestinal transit rate in the model group (MODEL) was markedly reduced (20.40 ± 3.67%) compared to the CON group (48.58 ± 2.02%, *p* < 0.001). When compared to the MODEL group, the low-dose group (LOW) showed an increase in small intestinal transit activity; however, this change did not reach statistical significance (*p* > 0.05). Conversely, the high-dose group (HIGH) exhibited a significantly improved small intestinal transit rate relative to the MODEL group (*p* < 0.001). Despite this enhancement, the transit rate in the HIGH group remained notably lower than that observed in the CON group (*p* = 0.015) (Figure 2).

### 3.4. Effects of Oat Fiber on Gastrointestinal Regulatory Peptides in Mice

**Figure 3:**
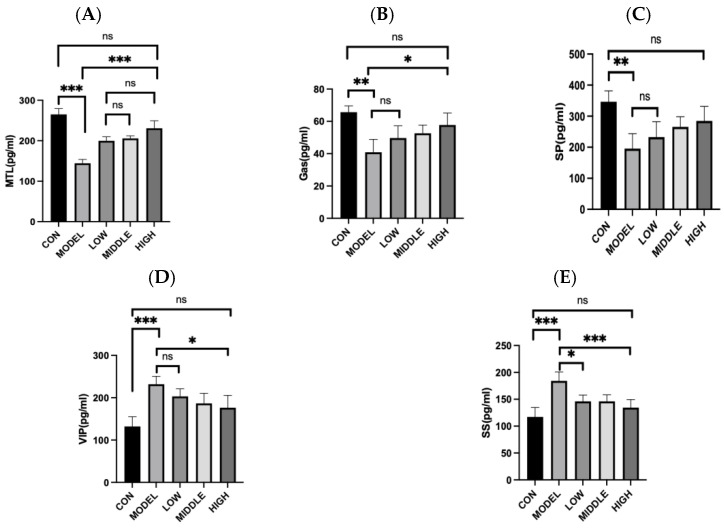
Effects of oat fiber on Gastrointestinal regulatory peptides in mice. (**A**) MTL; (**B**) GAS; (**C**) SP; (**D**) VIP; (**E**) SS. Note: *, **, ***, denoted *p* < 0.05, *p* < 0.01, *p* < 0.001, respectively. “ns” indicates that there was no significant difference.

Compared with the CON group, the serum motilin level in the MODEL group was markedly reduced (*p* < 0.001). In comparison to the MODEL group, oral administration of oat fiber led to a significant increase in motilin (MTL) levels in constipated mice (*p* < 0.001) (Figure 3A). When compared to the CON group, gastrin (GAS) levels in the MODEL group were significantly lower (*p* = 0.002). Following oral treatment with oat fiber, GAS levels in the LOW and MIDDLE groups showed an upward trend; however, these changes were not statistically significant (*p* > 0.05). Conversely, in the HIGH group of constipated mice, a significant increase in GAS level was observed after oat fiber administration (*p* = 0.036) (Figure 3B). Compared with the CON group, substance P (SP) levels in the MODEL group were significantly decreased (*p* < 0.01). After intragastric delivery of oat fiber, SP levels increased across all three dose groups, although the differences were not statistically significant (*p* > 0.05) (Figure 3C). In comparison to the CON group, vasoactive intestinal peptide (VIP) levels in the MODEL group were substantially elevated (*p* < 0.001). Compared with the MODEL group, following the oat fiber intervention, the HIGH group showed a significant reduction (*p* = 0.038). (Figure 3D). Compared with the CON group, the level of somatostatin (SS) in the MODEL group was markedly elevated (*p* < 0.001). Compared with the MODEL group, following oral administration of oat fiber, SS levels in the LOW and MIDDLE groups were significantly reduced (*p* < 0.05), while the HIGH group of constipated mice exhibited a highly significant decrease in somatostatin concentration (*p* < 0.001) (Figure 3E).

### 3.5. Effect of Oat Fiber on Serum 5-HT in Mice

**Figure 4:**
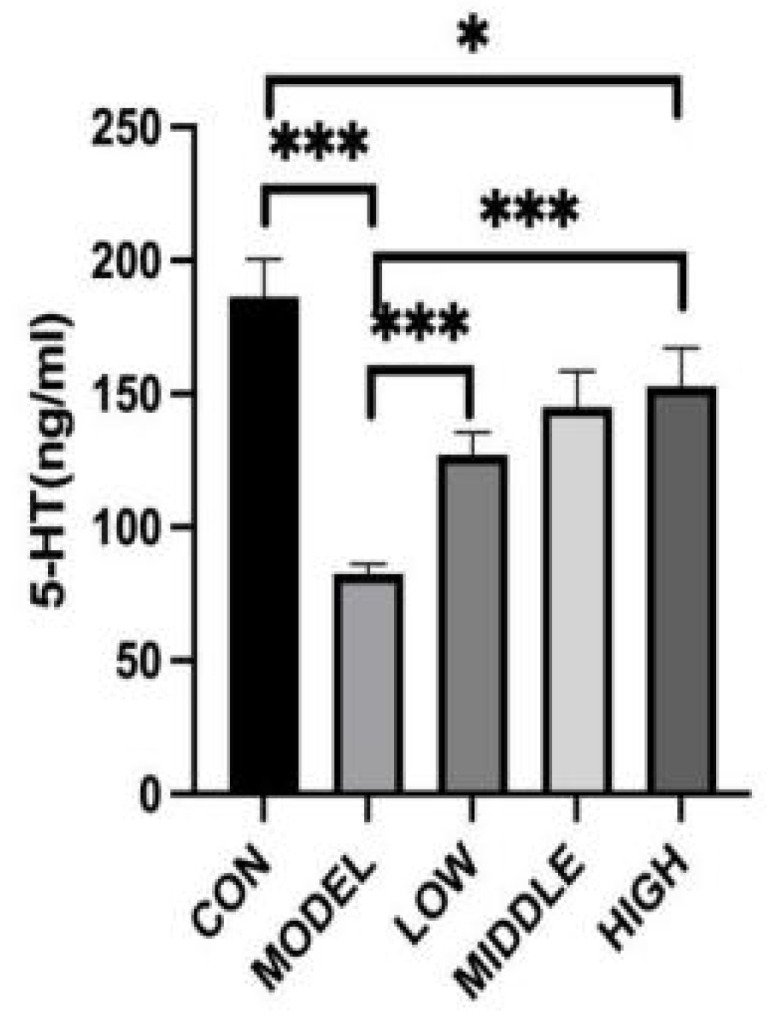
Effect of oat fiber on serum 5-HT in mice. Note: *, ***, denoted *p* < 0.05, *p* < 0.001, respectively.

The changes in serum 5-HT concentration in mice were presented in Figure 4. After continuous gavage with loperamide hydrochloride, the serum 5-HT concentration in the MODEL group was significantly decreased compared to the CON group (*p* < 0.001). After oral administration of oat fiber, the serum 5-HT levels in the three dose groups were markedly elevated compared to those in the MODEL group (*p* < 0.001). Nevertheless, when compared to the CON group, the 5-HT concentrations in these two groups were still significantly reduced (*p* < 0.001). Conversely, the serum 5-HT level in the HIGH group did not exhibit a statistically significant difference when compared to the control (CON) group (*p* = 0.021).

In this study, oat fiber increased serum 5-HT levels in constipated mice, potentially contributing to the regulation of gastrointestinal motility and alleviating constipation.

### 3.6. Oat Fiber Alleviated Intestinal Tissue Cell Damage and Colonic Tissue Inflammation in Constipated Mice

**Figure 5:**
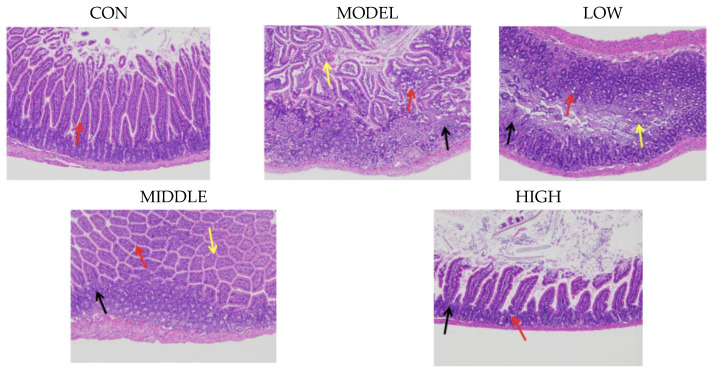
H&E staining of mouse colon.

Constipation could induce cell damage and immune inflammation in small intestine and colon tissues [11,12]. As shown in Figure 5, the overall structure of intestinal tissue in the CON group was normal, with regularly arranged intestinal villi and neatly aligned mucosal epithelial cells. No signs of atrophy or necrosis were observed in the intestinal villi or mucosal epithelial cells. The mucosal layer contained a rich number of goblet cells (indicated by red arrows), and no obvious congestion was observed in the mucosal layer. Additionally, there was no evident edema in the submucosa or inflammatory cell infiltration in the tissue.

In the MODEL group, the overall structure of the intestinal tissue was abnormal, with significant atrophy and shortening of intestinal villi (indicated by yellow arrows) and exfoliation and necrosis of mucosal epithelial cells. The quantity of goblet cells within the mucosal layer decreased (indicated by red arrows), and no obvious congestion was observed in the mucosal layer. However, loose edema was evident in the submucosa, and a significant infiltration of inflammatory cells was observed in the tissue (indicated by black arrows).

After intervention with oat fiber, the damage to intestinal mucosal epithelial cells in mice showed varying degrees of repair. Notably, in the HIGH group, the intestinal villi were regularly arranged, and the mucosal epithelial cells were neatly aligned, with no signs of atrophy, shortening, exfoliation, or necrosis. The mucosal layer contained a rich number of goblet cells (indicated by red arrows), and no obvious congestion or edema was observed in the mucosal or submucosal layers.

**Figure 6:**
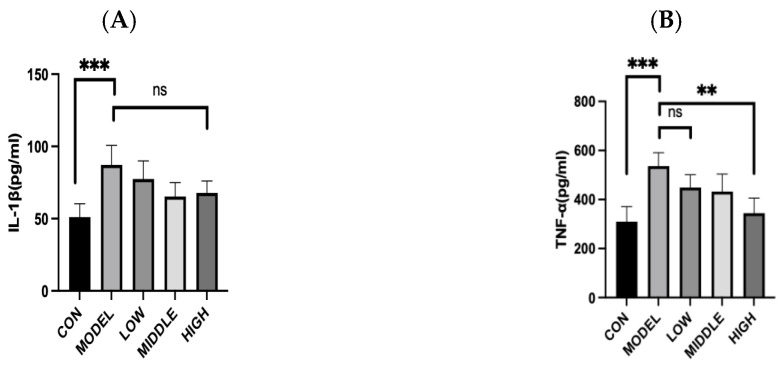
Inflammatory factor indicators in mice. (**A**) Relative transcription level of IL-1β. (**B**) Relative transcription level of TNF-α. Note: **, ***, denoted *p* < 0.01, *p* < 0.001, respectively. “ns” indicates that there was no significant difference.

As illustrated in Figure 6, the MODEL group exhibited significantly higher expression levels of both TNF-α and IL-1β compared to the CON group (*p* < 0.001), suggesting that constipation triggered an inflammatory reaction [13]. After oat fiber intervention, a reduction in the relative expression levels of TNF-α and IL-1β was noted, especially in the HIGH group, where a significant reduction in TNF-α expression was evident (*p* < 0.001).

In conclusion, gavage of oat fiber effectively alleviated intestinal barrier damage and immune-inflammatory responses induced by loperamide hydrochloride-induced constipation in mice.

### 3.7. Effect of Oat Fiber on Aquaporin Gene Transcript Levels in the Intestine of Constipated Mice

**Figure 7:**
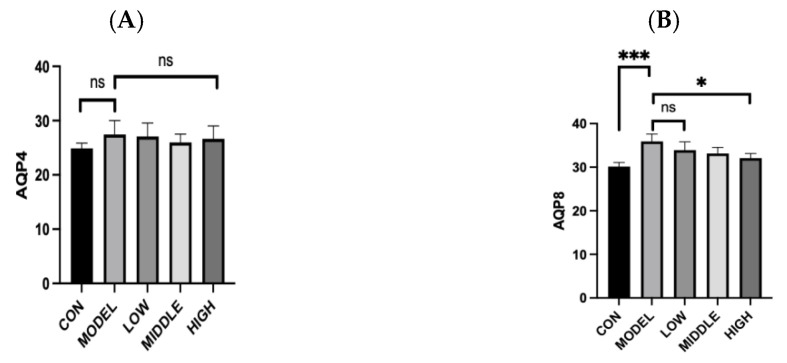
Mouse intestinal aquaporin gene transcript levels. (**A**): *AQP4* gene transcription level; (**B**): *AQP8* gene transcription level. Note: *, ***, denoted *p* < 0.05, *p* < 0.001, respectively. “ns” indicates that there was no significant difference.

As shown in Figure 7, the gene expression levels of Aquaporin 8 (AQP8) in the colon tissues of constipated mice were notably elevated compared to those in the control (CON) group (*p* < 0.001). And the transcriptional level of the AQP8 gene was significantly decreased after oat fiber intervention (*p* < 0.05). However, no significant differences in the gene expression levels of Aquaporins 4 (AQP4) were observed in the colonic tissues among the five groups of mice.

### 3.8. Effect of Oat Fiber on the Diversity of Gut Microbiota in Mice

#### 3.8.1. Alpha Diversity Analysis

**Figure 8:**
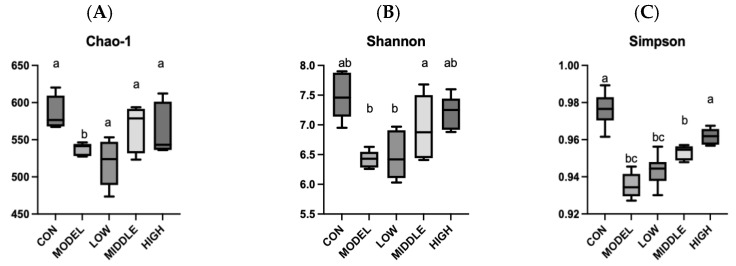
α-diversity of fecal microbiota in mice. (**A**) Chao-1 index; (**B**) Shannon index; And (**C**) Simpson index. Note: Different letters indicated that the difference was significant (*p* < 0.05).

Analysis of mouse gut microbiota were presented in Figure 8. In comparison to the control (CON) group, the Chao1, Shannon, and Simpson indices in the MODEL group were markedly reduced (*p* < 0.05). After gavage of oat fiber, the Shannon index and Simpson index of the intestinal flora in the HIGH group were significantly higher than those in the MODEL group (*p* < 0.01), while no significant difference was observed between the HIGH group and the CON group (*p* > 0.05). The results suggested that oat fiber could efficiently restore the microbial diversity in the intestines of constipated mice.

#### 3.8.2. Beta Diversity Analysis

**Figure 9:**
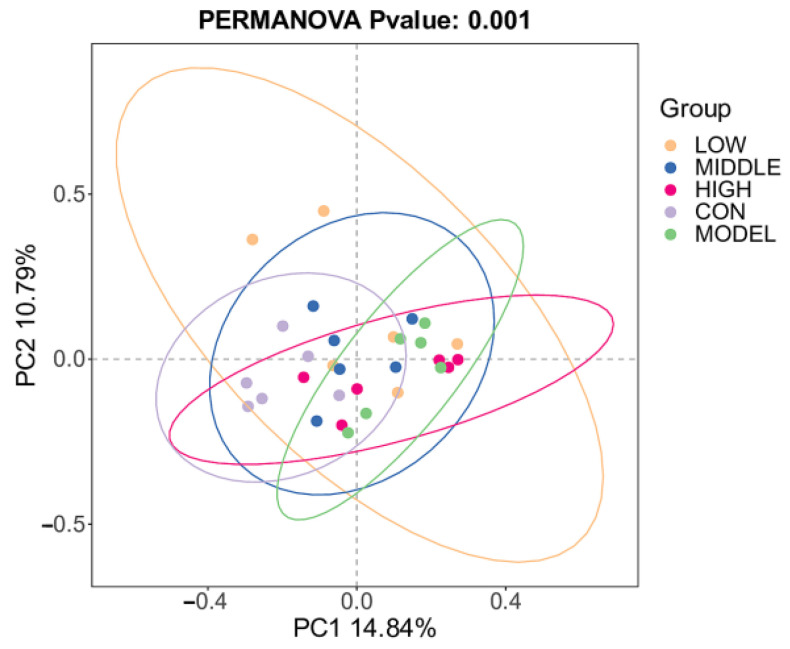
β-diversity of fecal microbiota in mice.

The results of β-diversity analysis of mouse gut microbiota were presented in Figure 9. PCoA analysis demonstrated distinct separation in the gut microbiota composition across all experimental groups (*p* < 0.001). Significantly, a distinct difference in the distribution of microbial communities was detected between the MODEL group and the control (CON) group (*p* < 0.05). The data points of the intervention groups (LOW, MIDDLE, HIGH) tended to cluster closer to those of the CON group, indicating a partial restoration of gut microbiota balance. However, significant differences still exist between the intervention groups and the CON group (*p* < 0.05). These findings suggested that oat fiber promoted the spontaneous regulation of gut microbiota balance in mice, as supported by previous studies [14,15,16].

#### 3.8.3. Analysis of Phylum Level

**Figure 10:**
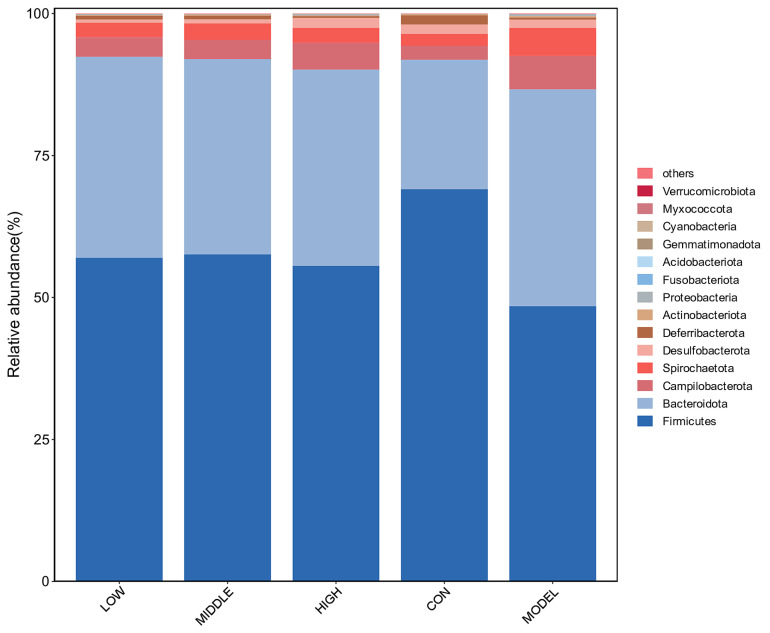
Phylum level analysis of intestinal microbiota in mice.

As shown in Figure 10, the top eight bacterial phyla with the highest abundance were: Firmicutes, Bacteroidetes, Campylobacterota, Spirochaetota, Desulfobacterota, and Deferribacterota. Among these, Firmicutes and Bacteroidetes were the dominant phyla, with their combined relative abundance exceeding 75%. This finding was consistent with previous studies [17,18,19]. Compared to the CON group, the relative abundance of Firmicutes was markedly reduced in the MODEL group (*p* = 0.012), whereas Bacteroidetes showed a significant increase (*p* = 0.044). Following treatment with oat fiber, the proportion of Firmicutes was notably restored, suggesting that oat fiber has the potential to modulate gut microbiota and enhance the intestinal microenvironment in constipated mice.

Numerous studies have reported significant variations in the proportional representation of Firmicutes and Bacteroidetes in the gut microbiota of constipated mice compared to that of healthy control animals. These differences might be influenced by factors such as the source of the mice, living environment, dietary intake, and experimental conditions. This study further confirmed that oat fiber could effectively regulate intestinal flora and improve the intestinal microecological environment in constipated mice [20].

#### 3.8.4. Analysis of Genus Level

**Figure 11:**
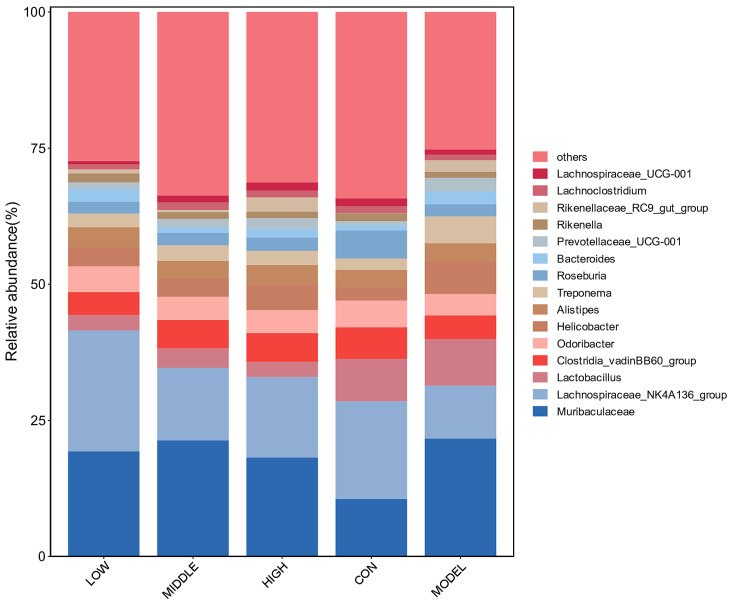
Genus level analysis of intestinal flora in mice.

As shown in Figure 11, the top 15 bacteria genera with the largest abundance were: Muribaculaceae, Lachnospiraceae_NK4A136_group, Lactobacillus, Clostridia_vadinBB60_group, Odoribacter, Helicobacter, Alistipes, Treponema, Roseburia, Bacteroides, Prevotellaceae_UCG-001, Rikenella, Rikenellaceae_RC9_gut_group, Lachnoclostridium, Lachnospiraceae_UCG-001, among which, there was a significant difference in Lachnospiraceae_NK4A136_group in the intestinal flora of mice (*p* < 0.05), which belonged to Lachnospiraceae, which had a positive effect on intestinal health [21,22]. Compared with the CON group, Lachnospiraceae_NK4A136_group was significantly decreased in the MODEL group, which was significantly increased after oat fiber intervention. In addition, Roseburia abundance was also significantly decreased in the MODEL group, which was consistent with previous findings [23]. After oat fiber intervention, the abundance of this genus was increased. In conclusion, oat fiber could regulate intestinal flora and improve intestinal microecological environment in mice with constipation.

#### 3.8.5. Correlation Analysis of Intestinal Flora with Serum Indexes and Intestinal Aquaporins

**Figure 12:**
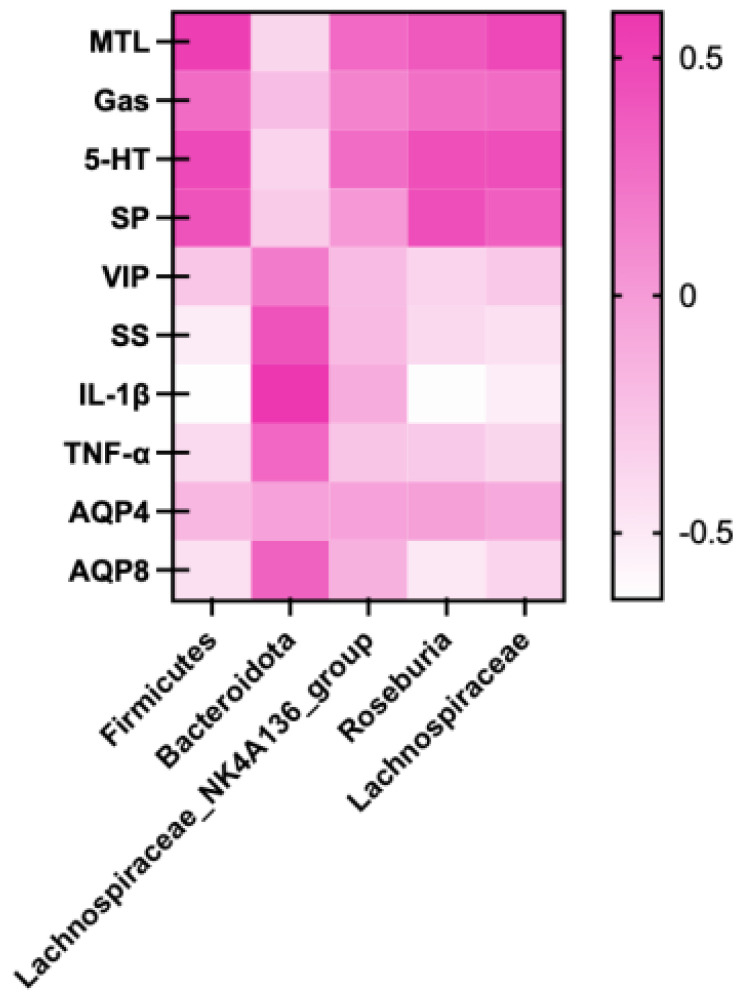
Spearman correlation coefficient heat map of the difference between gut microbiota and serum indexes and intestinal aquaporin indexes of mice. Note: Color indicated positive and negative correlation coefficients, red indicated positive correlation, which indicated negative correlation, and darker color indicated stronger correlation.

To further investigate whether the improvement in serum gastrointestinal peptides, inflammatory factors, and intestinal aquaporin indices in constipated mice was associated with changes in gut microbiota, the Spearman method was employed to analyze the correlations between these functional indicators. The results are presented in Figure 12. Significant correlations were observed between several bacterial taxa (Firmicutes, Bacteroidetes, Roseburia, Lachnospiraceae) and multiple functional indicators (*p* < 0.05). Specifically, Firmicutes exhibited significant positive correlations with MTL and 5-HT levels (*p* < 0.05), while showing significant negative correlations with SS, IL-1β, TNF-α, and AQP4 levels (*p* < 0.05). Bacteroidetes exhibited a statistically significant positive association with IL-1β concentrations (*p* < 0.05), along with significant negative correlations with 5-HT and SP levels (*p* < 0.05). Roseburia was positively linked to MTL, 5-HT, SP, and AQP8 levels (*p* < 0.05), and negatively associated with SS, IL-1β, and AQP4 concentrations (*p* < 0.05). Lachnospiraceae showed positive correlations with MTL and 5-HT levels (*p* < 0.05), and a significant negative relationship with IL-1β concentrations (*p* < 0.05). And correlations between gut microbiota and other indicators were tested using Spearman’s rank correlation. All *p*-values were corrected for multiple testing using Benjamini-Hochberg FDR (*q* < 0.05) to identify statistically significant associations.

## 4. Discussion

In this study, oat fiber was used to alleviate loperamide-induced constipation in mice, and the underlying mechanisms were explored. The results demonstrated that oat fiber effectively alleviates constipation symptoms.

Patients with constipation often exhibited dry and hard stools, which resulted from low fecal water content and could cause defecation difficulty or even bowel wall damage. Therefore, improving fecal status was a critical task in constipation treatment. Loperamide hydrochloride, as a peripheral opioid receptor agonist, inhibited intestinal peristalsis and fluid secretion [24]. Oat fiber significantly increased fecal water content in mice, indicating its high water retention capacity and its ability to improve intestinal water secretion and reabsorption as well as stool consistency. The first black stool time reflected the total transit time of digesta from the stomach to the anus and subsequent excretion, which indicated overall gastrointestinal peristalsis ability. A longer first melena time suggested weaker gastrointestinal propulsion. In this study, oat fiber intervention significantly reduced the first black stool time and improved the small intestine propulsion rate, consistent with previous findings [25].

Aquaporins (AQPs) are a class of cell membrane proteins that regulate the transport of water and certain small molecules. Previous studies have identified AQPs as functioning as flow-regulating valves within cells, enabling the selective absorption and secretion of water molecules to maintain intestinal fluid homeostasis [26]. The transcription levels of Aquaporins 8 (AQP8) genes in the colon of constipated mice were significantly higher than those in the control group, consistent with the results reported by Li et al. [26] suggesting that elevated AQP gene transcription promoted intestinal water absorption, leading to dry stools. However, no effects of Aquaporins 4 (AQP4) in colonic tissue were observed in this study. In future studies, we intend to conduct an in-depth investigation into the signaling pathways associated with the functional regulation of intestinal aquaporins. We will also conduct a comprehensive and thorough investigation of the other types of proteins.

MTL promotes gastric contraction and facilitates segmental motility in the small intestine, thereby enhancing intestinal transit efficiency through periodic segmentation movements [27]. GAS played a key role in regulating gastric acid secretion and stimulating digestive tract activity [28]. SP was an excitatory neuropeptide associated with increased intestinal peristalsis, and low levels of SP were linked to the occurrence of constipation [29]. VIP was an important regulator of intestinal peristalsis that relaxed gastrointestinal smooth muscles [30]. SS was an inhibitory neurotransmitter that suppressed the release of SP, relaxed gastrointestinal smooth muscles, reduced gastrointestinal peristalsis, and increased gastrointestinal transit time [31].

5-HT influenced intestinal motility both directly and indirectly via neurons. It played a crucial role in regulating gastrointestinal motility, smooth muscle contraction, intestinal secretion, and nerve signal transduction [32]. An increase in 5-HT concentration could induce the release of SP and excitatory neurotransmitters, thereby enhancing intestinal peristalsis. A decrease in 5-HT concentration promoted the release of inhibitory neurotransmitters such as nitric oxide (NO), leading to reduced gastrointestinal motility [33]. Additionally, under inflammatory conditions, 5-HT exhibited both anti-inflammatory and pro-inflammatory effects on the intestinal tract by binding to different receptors [34]. Constipation altered the content of gastrointestinal active peptides, affecting intestinal smooth muscle contraction and relaxation and inhibiting peristalsis through neurotransmitter regulation such as choline, monoamines, and peptides. This aligned with our findings that oat fiber enhanced intestinal peristalsis in mice. After binding to receptors in the gastrointestinal tract, 5-HT influenced intestinal transport; thus, serum 5-HT concentration reflected intestinal function change [35]. Constipation symptoms were associated with decreased 5-HT concentrations. Increasing 5-HT concentration induced the release of SP and excitatory neurotransmitters, thereby promoting intestinal peristalsis, consistent with our findings [36]. In future research, we will perform a more focused analysis on the brain-gut axis and specifically examine the effects of neurotransmitters on hormones and other related biochemical substances.

Long-term consumption of oats may confer potential benefits in the context of inflammatory bowel diseases. Gao et al. [37] found that constipation was accompanied by intestinal inflammation, which were also observed in our study, including manifestations such as colon infiltration by inflammatory cells, disruption of intestinal tissue structure, villus atrophy and shortening, shedding and necrosis of mucosal epithelial cells, and a marked decrease in goblet cell numbers. These phenomena were suppressed following oat fiber intervention. Gao et al. [38] further demonstrated that oat fiber exhibited anti-inflammatory properties and reduced intestinal permeability, consistent with our results. Furthermore, this study validated the involvement of Firmicutes, Bacteroidetes, and Lachnospiraceae in relieving constipation, indicating that oat fiber regulated gut microbiota and improved intestinal microecology in constipated mice.

In this study, we specifically investigated the effect of oat fiber on the gut microbiota of constipated mice. Future research will focus on analyzing additional aspects of the intestinal microecology, such as short-chain fatty acids (SCFAs). By employing a metabolomics approach, a comprehensive analysis could be conducted on metabolites that might alleviate constipation. These metabolites could be correlated with detected physiological and biochemical indicators or changes in the microbiota composition, thereby providing a more thorough understanding of how dietary fiber alleviated constipation across various dimensions. While the pathophysiological mechanisms and symptoms of loperamide hydrochloride-induced constipation in mice resembled those of clinical constipation patients, certain differences still existed. In future studies, population-based investigations could further validate the effects and mechanisms of dietary fiber in alleviating constipation symptoms. In addition to their application in animal models of constipation, oats exhibit antioxidant properties and demonstrate the ability to modulate postprandial glucose levels, blood pressure, cholesterol, and other clinically relevant parameters [39,40]. Based on these findings, further investigations into the physiological effects of oats can be conducted.

Furthermore, a limitation of this study is the absence of an oat fiber group without loperamide, which did not allow for excluding the potential influence of loperamide. Therefore, this issue will be addressed in future experiments through more comprehensive study designs.

## 5. Conclusions

In conclusion, regarding chemical barriers, oat fiber could enhance intestinal fluid secretion and promote intestinal peristalsis by regulating the expression of intestinal aquaporins and facilitating neurotransmitter signaling. In terms of the immune barrier, oat fiber could protect the body from pathogen invasion by repairing the intestinal mucosa and modulating the release of inflammatory factors in intestinal immune cells to alleviate the inflammatory response. With respect to biological barriers, oat fiber could maintain intestinal environmental homeostasis by regulating the composition of the gut microbiota. Therefore, oat fiber demonstrated advantages in repairing the intestinal barrier, as evidenced by its ability to regulate aquaporin expression, inhibit the release of pro-inflammatory factors, and enhance gut microbiota diversity.

## Figures and Tables

**Table 1:** Reverse transcription reaction system.

Component	Volume
5 × SweScript All-in-One SuperMix for qPCR	4 μL
gDNA Remover	1 μL
Total RNA *	10 μL
Nuclease-Free Water	Add to 20 μL

*: Total RNA refers to the complete pool of RNA molecules extracted from a cell or tissue, representing all RNA species present under a specific physiological or experimental condition.

**Table 2:** Primer sequences.

Target Gene	Primer Sequence (5′−3′)	
	Forward	Reverse
AQP4	CAGCATCGCTAAGTCCGTCT	GACTCCCAATCCTCCAACCA
AQP8	GAATAGTCCGAATACTGGGCTCCT	GTTGAAGTGTCCACCGCTGATG

**Table 3:** Effects of oat fiber on body weight of mice (g).

Groups	Before Modeling	After Modeling	After Intervention
CON	23.13 ± 0.61 ^a^	25.53 ± 0.73 ^a^	27.37 ± 1.08 ^a^
MODEL	22.68 ± 0.80 ^a^	21.32 ± 0.88 ^b^	18.82 ± 1.39 ^b^
LOW	23.32 ± 1.14 ^a^	22.10 ± 1.19 ^b^	23.37 ± 1.33 ^c^
MIDDLE	22.92 ± 0.58 ^a^	21.92 ± 0.56 ^b^	23.98 ± 0.39 ^c^
HIGH	23.20 ± 0.72 ^a^	22.08 ± 0.73 ^b^	24.83 ± 0.76 ^c^

Note: Comparison within the same column. Different lowercase letters represented significant differences (*p* < 0.05).

**Table 4:** Effect of oat fiber on defecation in mice.

Groups	Number of Fecal Pellets (Pellets/3 H)	Fecal Moisture Content (%)	Time to First Black Stool (min)
CON	20.17 ± 2.23 ^a^	60.17 ± 1.21 ^a^	140.33 ± 6.56 ^d^
MODEL	3.50 ± 1.05 ^c^	40.33 ± 2.78 ^d^	301.83 ± 20.61 ^a^
LOW	10.67 ± 2.50 ^b^	48.45 ± 0.79 ^c^	230.33 ± 14.25 ^b^
MIDDLE	13.50 ± 2.26 ^b^	50.25 ± 1.13 ^c^	214.67 ± 4.72 ^b^
HIGH	14.67 ± 2.50 ^b^	55.59 ± 0.56 ^b^	193.50 ± 4.85 ^c^

Note: Comparison within the same column. Different lowercase letters represented significant differences (*p* < 0.05).

## Data Availability

All raw data supporting the reported results are available from the authors on request.
